# Archimedes’ law explains penetration of solids into granular media

**DOI:** 10.1038/s41467-018-03344-3

**Published:** 2018-03-16

**Authors:** Wenting Kang, Yajie Feng, Caishan Liu, Raphael Blumenfeld

**Affiliations:** 1 State Key Laboratory of Turbulence and Complex System, College of Engineering, Peking University, 100871 Beijing, China; 2 Imperial College London, London SW7 2AZ, UK; 3 University of Cambridge, Cambridge CB3 0HE, UK; 4 National University of Defence Technology, Changsha, Hunan 410073, China

## Abstract

Understanding the response of granular matter to intrusion of solid objects is key to modelling many aspects of behaviour of granular matter, including plastic flow. Here we report a general model for such a quasistatic process. Using a range of experiments, we first show that the relation between the penetration depth and the force resisting it, transiently nonlinear and then linear, is scalable to a universal form. We show that the gradient of the steady-state part, *K*_*ϕ*_, depends only on the medium’s internal friction angle, *ϕ*, and that it is nonlinear in *μ* = tan *ϕ*, in contrast to an existing conjecture. We further show that the intrusion of any convex solid shape satisfies a modified Archimedes’ law and use this to: relate the zero-depth intercept of the linear part to *K*_*ϕ*_ and the intruder’s cross-section; explain the curve’s nonlinear part in terms of the stagnant zone’s development.

## Abstract (web summary)

The penetration dynamics of solid objects into granular media has been described by theories that are constrained to the use of phenomenological models or empirical parameters. Here, Kang et al. propose and test a parameter-free model for the dependence of the resistance force on penetration depth.

## Introduction

The ubiquity of dense granular matter in nature and the important role it plays in human society cannot be overestimated. Its significance focused much attention for centuries, but research into the fundamental science of granular matter underlying its rich behaviour exploded in the past couple of decades. In spite of the intensive activities, both the statics and dynamics of granular matter are not well understood with many remaining open problems^1,2^.

A canonical problem in the field is the modelling of the penetration dynamics of a large object within a granular material made of much smaller, but macroscopic particles. This problem has been receiving growing attention in recent years for its multiple applications, e.g., locomotion of terrestrial animals^3,4^, robots working on granular substrates^5–7^, and crater formation in geological and astrophysical fields^8–10^. Studies on this problem also play significant roles in understanding static properties of granular materials^11,12^, characterising the dynamics of granular flows^13,14^, and understanding the response of perturbed granular materials, such as shear banding^15,16^, sinking effect^17^, and the jamming transition^18–21^.

The penetration dynamics is modelled usually using macroscopic laws of drag force exerted on the intruder^22–24^, which describe the drag force as a combination of a hydrostatic-like force *F*_*z*_ and a viscous force *F*_v_. The hydrostatic-like force *F*_*z*_ results from the frictional plasticity of the granular matter^22,25^, and the viscous force *F*_v_ results from momentum transfer between the grains^26,27^. To bridge between the developing knowledge of static granular matter and its dynamic flow, we must understand and construct a predictive model for the penetration process under quasistatic conditions. Advancing such an understanding is the main aim of this paper.

A number of experiments demonstrated that the force, *F*_*z*_, is hydrostatic-like, varying linearly with penetration depth *h* for submerged or flat-bottom intruders^5,25^. Based on this observation, an empirical resistive force theory (RFT)^3^ has been proposed for the resistance force on objects intruding granular media quasistatically at different orientations. This approach proved adequate to model the kinematics of slow-moving locomotors^3,6,28^. It also agrees with a local friction force model (LFFM), recently proposed in ref. ^23^

1 $${\rm d}{\bf{F}} = K_\phi P{\rm d}{\bf{S}} = k\mu P{\rm d}{\bf{S}}.$$

In this relation, d**S** is an infinitesimal area element pointing normal to the intruder surface, *P* = *ρ*_s_*gh* is the hydrostatic-like pressure proportional to the packing density *ρ*_s_ of the granular matter, *K*_*ϕ*_ is a coefficient (>1) proportional to the internal friction coefficient *μ* via a fitting parameter *k*.

Both RFT and LFFM are based on the assumption that the friction of the granular tangential flow against the intruder’s surface contribute negligibly to the resistance force. Askari and Kamrin^29^ then showed by FEM simulations, in which the granular medium is modelled as a continuum of bulk density *ρ*_s_ and internal friction coefficient *μ*, that these features are a consequence of two properties: cohesionless and a friction yield criterion.

Yet, existing models for the quasistatic resistance force have two main deficiencies: they are mainly phenomenological, thus providing little understanding of the physics of the penetration process, and the parameters governing the force magnitude need to be determined experimentally, undermining the model’s predictive power. It is essential to develop a physics-based quantitative model for the quasistatic resistance force in cohesionless dry granular matter.

Here, we address this issue both theoretically and experimentally. First, we carry out experiments to measure the resistance force on cylinders penetrating vertically into five different granular materials. By varying the bottom area and shape of the intruders, we find a unified dimensionless pressure–depth curve. This curve consists of an initial nonlinear segment, extending to a similar depth (when properly scaled) for all experiments, corresponding to a compression of the granular material ahead of the advancing intruder, which gives rise to formation of a stagnant zone (SZ). This transient is followed by an extended linear region, whose gradient depends only on the medium’s internal friction angle *ϕ*. This region suggests that the medium yields in a fluid-like manner. We then analyse the process, treating the granular matter as a continuum at a critical state, characterised by parameters *ρ*_s_ and *ϕ*. We conjecture that the initial penetration process gives rise to a conical SZ below the cylinder, caused by shear jamming^5,29^. Using the Mohr–Coulomb (MC) yield criterion and the method of characteristics, we model the steady state of the conical SZ and obtain theoretically an explicit expression for the curve’s gradient. This gradient is found to be independent of the intruder’s geometry and it provides the coefficient *K*_*ϕ*_ in Eq. (1). However, we find that this coefficient is not linear in *μ* = tan *ϕ*, casting doubt on Eq. (1). This leads us to conclude that the quasistatic force on any cylinder, whether its bottom is flat, conical or half spherical, can be described universally by Archimedes’ law, albeit scaled by the factor *K*_*ϕ*_ > 1, which we calculate. We then show that the Archimedes’ law can explain the force–depth relation, including the dependence of the constant term on medium nature and intruder cross-section area, as well as the effective steady-state size of the SZ.

## Results

### Experiments

**Fig. 1:**
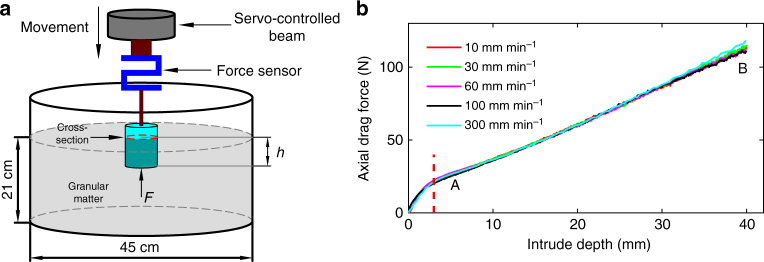
Experimental setup and validation of the quasistatic regime. **a** Sketch of the experimental apparatus. An intruder is connected to the servo-controlled beam through a force sensor. The intruder is inserted into the granular medium at constant low velocity, while the vertical displacement and total vertical resistance force are continuously recorded. **b** The raw data for the vertical resistance force on a cylindrical of diameter *D* = 40 mm and length *L* = 50 mm, penetrating quartz sand with different velocities, ranging from 10 mm min^−1^ to 300 mm min^−1^. The independence of the velocity indicating the quasistatic state of the experiment. The red dashed line marks the end of the transient nonlinear regime

**Fig. 2:**
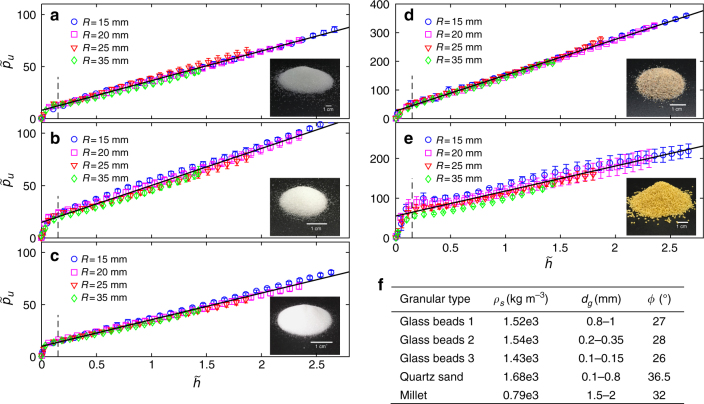
Dimensionless pressure–depth curves for the different granular materials. **a**–**c** Glass beads 1–3, respectively; **d** quartz sand; **e** millet. The error bars represent standard deviations over samples, as detailed in the Methods section. Photos of the respective granular materials are shown in the lower right corners. **f** The parameters for the five granular materials: *ρ*_s_ is the packing mass density, *d*_g_ is the particle diameter and *ϕ* is the angle of repose, accurate to ±2°. The plots collapse very well for all the granular media, regardless of the intruder size. The nonlinear-to-linear crossover, shown by a vertical dashed line, is at a similar value in all the experiments: $\tilde h_0 = 0.15 \pm 0.06$. The solid lines are linear fits to the experimental data for $\tilde h > 0.15$. The fit quality is: *R*^2^ >0.990, 0.977, 0.985, 0.996 and 0.935, respectively, for the glass beads types 1–3, the sand and the millet

The experimental apparatus is sketched in Fig. 1a. A cylindrical intruder was connected to a servo-controlled beam through a force sensor that records the total axial force opposing the downward motion. The intruder is pushed into a barrel of diameter 45 cm, filled with granular material up to a height of 21 cm. Throughout the process, the cylinder was kept well away from the barrel’s boundaries, eliminating boundary effects^30,31^. For generality, several granular materials were used: three kinds of glass beads, made of different particle size distributions, dry quartz sand, and millet. Unlike the glass beads, the quartz sand grains are much more angular and irregular, while the millet grains are softer and less spherical. The physical parameters of these materials are shown in Fig. 2f, where the internal friction angle *ϕ* refers to the angle of repose, accurate to ±2°.

In the first set of experiments, we used four aluminium cylinders of different radii, *R* = 15, 20, 25, and 35 mm, but the same length *L* = 50 mm. The penetration depth, *h*, was limited to be below *L* to avoid both end effects at the top of the cylinder and jamming effects close to the bottom of the barrel. To ensure velocity independent behaviour, the penetration velocity was restricted to below a critical value^32^, $v_{\rm c} = \sqrt {2gd_{\rm g}} {{\rm /}}10$, where *g* is gravity acceleration and *d*_g_ is the mean diameter of granules. This was tested by measuring the force on a *R* = 20 mm cylinder penetrating into sand at velocities ranging from 10 mm min^−1^ to 300 mm min^−1^. Indeed, Fig. 1b verifies that the resultant force–depth curves are hardly different. The velocities were then limited to below 30 mm min^−1^ in all experiments.

The experiments reported in refs. ^5,23^ demonstrated that the friction between the intruder surface and grains is negligible relative to the force required to push material below the cylinder out of the way. This allowed us to define a mean pressure, *p*_*u*_ = *F*/*S*, where *F* is the measured vertical resistance force and *S* is the cylinder’s cross-section area. To non-dimensionalise our results, as well as for reasons to be understood below, we scaled the mean pressure, $\tilde p_u = p_u{{\rm /}}\left( {\rho _{\rm s}gR} \right)$, and the penetration depth, $\tilde h = h{{\rm /}}R$.

Figure 2a–e shows the dependence of $\tilde p_u$ on $\tilde h$ for the five granular materials, when penetrated by the four different cylinders. For each medium, the curves collapse onto a master curve for all the different intruders. This result agrees with the surface-level superposition rules postulated by RFT^3^ and LFFM^23^.

All the $\tilde p_u - \tilde h$ curves consist of an initial nonlinear regime, $\tilde p_u = f_1\left( {\tilde h \le \tilde h_0} \right)$, corresponding to material compression ahead of the intruder, which culminates in a fully formed rigid SZ (see below), followed by a much longer linear regime, in which $\tilde p_u\sim \tilde h$. The nonlinear-to-linear crossover was determined, by inspection, to be at $\tilde h_0 = 0.15 \pm 0.06$. Identifying $\tilde p_u\left( {\tilde h_0} \right)$ as the crossover pressure to the steady state, we define the gradient of the linear region beyond this point as *K*_*ϕ*_. The dimensionless pressure–depth relation is then

2 $$\tilde p_u = \left\{ {\begin{array}{*{20}{c}} {f_1(\tilde h)} & {\tilde h \le \tilde h_0} \\ {\tilde p_0 + K_\phi \tilde h} & {\tilde h > \tilde h_0} \end{array}} \right.$$

Similar behaviours were also observed in previous resistance force measurements^5,24,27,33–35^. Particle image velocimetry measurements^5,36,37^ and discrete element method simulations^38^ showed that, during the transient nonlinear regime, a conical SZ forms under the flat bottom of intruder segment, driven by a local shear jamming process^19^. This region then advances as a rigid cone ahead of the intruder^39,40^. While the cone formation process is complex and not fully understood^5,40^, this analysis provides a way to model it in terms of the intruder’s cross-section area and the location of the crossover depth to steady state (see below).

The conical SZ advances ahead of the intruder at the same downward speed, parting the medium and wedging matter sideways^5,8,34^. This means that the quasistatic resistance force on the intruder is governed by the intergranular contact friction at the cone advancing surface^23^. It follows that the slope of the linear part, *K*_*ϕ*_, should depend on *ϕ*. In our experiments, the value of *K*_*ϕ*_ ranges very widely for different granular materials: from about 20 to more than 100.

**Fig. 3:**
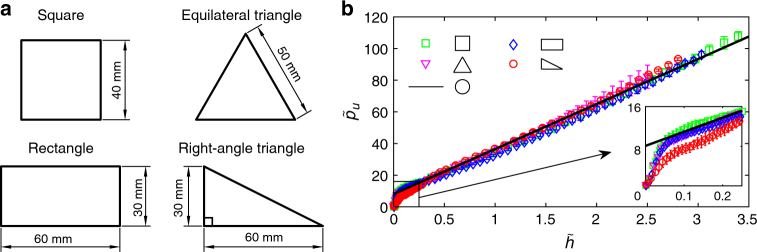
Independence of the force–depth curve of the intruder’s cross-section shape. **a** The cross-sections, normal to the direction of penetration, of the polygonal prisms used in the experiments. **b** Dimensionless pressure–depth curves for different polygon prisms penetrating glass beads 1. The black line is the fit in Fig. 2a; inset: a zoom in on the nonlinear regime. $\tilde h$ is measured in units of the equivalent radius, $R_{\rm e} = \sqrt {S{\mathrm{/}}\pi }$. The measurements were taken on 3 samples for each shape and the error bars represent the standard deviation over the samples. The fits qualities are: *R*^2^ >0.997, 0.992, 0.998, and 0.993, respectively, for the square, rectangle, equilateral triangle and right-angle triangle. The experimental data of all the polygon intruders collapse on the fitted curve in Fig. 2a for all the cylinders (solid line), suggesting the *K*_*ϕ*_ is a pure material constitutive parameter

To test whether *K*_*ϕ*_ depends on intruder properties or is a pure material constitutive parameter, we carried out a suite of penetration experiments with flat-bottom prisms of four cross-section shapes: square, equilateral triangle, rectangle, and right-angled triangle, shown in Fig. 3a. The prisms were made of aluminium and *L* = 120 mm long. The granular medium was the type 1 glass beads. The depth and pressure scaling depend on the intruder’s cross-section area, *S* and we define an equivalent radius $R_{\rm e} = \sqrt {S{{\rm /}}\pi }$. This scaling indeed leads to a collapse of the steady state of all the pressure–depth curves onto a unique master curve, regardless of cross-section shape, as shown in Fig. 3b. Moreover, this curve is identical to the one for the cylindrical intruder. This establishes that *K*_*ϕ*_ is indeed independent of the shape and size of the intruder and, therefore, that it is a constitutive characteristic of the granular material^23,34^. In the next section, we present a theoretical model to predict this parameter. Moreover, there is also a good collapse in the nonlinear regime when the aspect ratio of the cross-sections is not too different than 1 (see Discussion below).

### Theoretical analysis

**Fig. 4:**
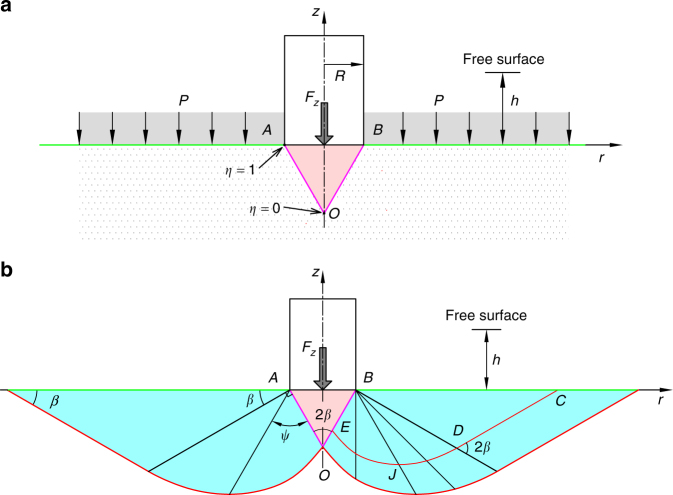
The theoretical model. **a** The system includes the intruder and a conical SZ ahead of it. **b** Examples of the “+” (red lines) and “−” (black lines) characteristic paths in the *rz* plane, as well as their dependence on the angle *β* = *π*/4 − *ϕ*/2. *α* = 0 at the intruder’s bottom and −*π*/2 at the cone surface

Quasistatically yielding cohesionless dry granular media can be modelled as a continuum of bulk density *ρ*_s_ and internal friction angle *ϕ*^29^. To model the stress field, the mechanical equilibrium conditions are closed by a yield criterion, for which there are several models. We choose here to use the MC criterion^41^, $\left| {\tau {{\rm /}}\sigma _{\rm n}} \right| = \mu \equiv {{\rm tan}}{\kern 1pt} \phi$, where *τ* is the local shear stress and *σ*_n_ is the corresponding normal stress. Several experiments established that the penetration process gives rise to a conical SZ ahead of the intruder, advancing as a rigid body^36–38,42^. Taking this into consideration, the boundary conditions are sketched in Fig. 4a and include the downward pressure at depth *h* and the resistive force, *F*_*z*_, on the intruder. We assume that the free surface, left behind, is flat, which is a good assumption as long as any deviation from flatness is appreciably smaller than the size of the SZ. Supplementary Fig. 1 supports this assumption.

Expressing the stress components (Supplementary Fig. 2) in terms of the mean of the major and minor principal stresses, *σ*_0_, and the angle between the major principal stress and the radial direction, *α*, gives a hyperbolic set of equations in these two variables. The equations can be solved by characteristics (Supplementary Note 1 and Fig. 3), which are paths in the plane along which they reduce to two ordinary differential equations^43^. The solution yields two families of characteristics, whose local gradients are

3 $$\kappa _ \pm = {\rm d}z{{\rm /}}{\rm d}r = {{\rm tan}}\left( {\alpha \pm \beta } \right)\quad ;\quad \beta = \pi {{\rm /}}4 - \phi {{\rm /}}2.$$

Conveniently, the angle between two + and − characteristic curves, passing at any one point in the *rz* plane, is always 2*β*. Focusing on the steady state, when the SZ is fully formed, and noting that *α* = −*π*/2 everywhere on the cone surface, we can solve for *σ*_0_ along a characteristic path:

4 $$\sigma _0 = \frac{P}{{1 - {{\rm sin}}{\kern 1pt} \phi }}A(\eta ,\phi ){{\rm e}}^{\pi {\kern 1pt} {{\rm tan}}{\kern 1pt} \phi },$$

where 0 ≤ *η* ≤ 1 parameterises the cone surface and runs from its apex to its base (see Fig. 4b) and *A*(*η*, *ϕ*) is derived in the Methods section. This analysis is in good agreement with the observations in refs. ^36–38,42^.

Obtaining *σ*_*z*_ from *σ*_0_ and integrating it along the cone surface to obtain *F*_*z*_ (Supplementary Note 2), we finally identify the coefficient of *h*:

5 $$K_\phi = \frac{{2(1 + {{\rm sin}}{\kern 1pt} \phi )}}{{(1 - {{\rm sin}}{\kern 1pt} \phi )}}{{\rm e}}^{\pi {\kern 1pt} {{\rm tan}}{\kern 1pt} \phi }{\int}_{0}^{1} {\kern 1pt} \eta A(\eta ,\phi ){\rm d}\eta .$$

**Fig. 5:**
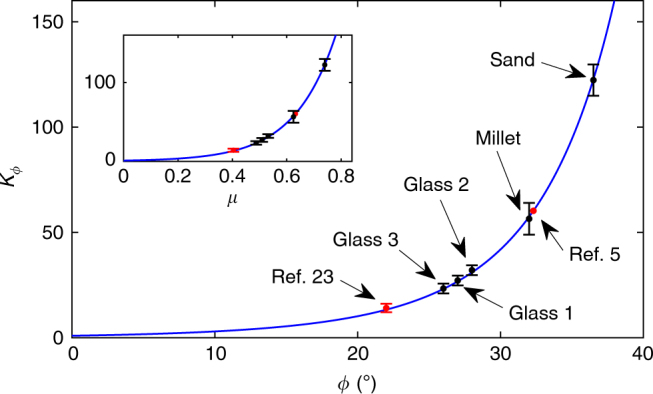
Validation of the calculation of *K*_*ϕ*_. *K*_*ϕ*_, calculated from Eq. (5) (solid line), and its measured values. The black marks, from all our experiments, and the red mark, from refs. ^5,23^, fall squarely on the predicted curve. Error bars represent one standard deviation about the mean. All our experimental values (black points) incur a horizontal error bar of ±2°, which was omitted to avoid clutter. Inset: the relationship between *K*_*ϕ*_ and *μ* = tan*ϕ* is clearly superlinear, challenging the assumption of linearity in the LFFM. The error bars represent one standard deviation about the mean. Using the data reported in ref. ^5^, we estimate the internal friction angle of their poppy seed granules as 32.3° ± 0.3°. The quality of the fit between the experimental points and the theoretical curve is *R*^2^ = 0.996

This result is significant. It shows that: (i) *K*_*ϕ*_ depends only on *ϕ* and on no property of the intruder; (ii) *K*_*ϕ*_ is not linear in tan* ϕ*, in contrast to the proposed relation (1)^23^. In Fig. 5a, we plot the values of *K*_*ϕ*_, computed numerically from Eq. (5), as a function of *ϕ* and compare them with the experimental measurements for the five granular media. The agreement between the model prediction and the experimental values is excellent. From the data reported in ref. ^23^ for glass beads with *ϕ* = 22°, we calculate *K*_*ϕ*_ = 13.36. This also agrees very well with their measured result, which is equivalent to *K*_*ϕ*_ = 14 ± 2. There is also a good agreement with ref. ^5^, in which force–depth data are reported for intrusion of ‘foot’ objects into assemblies of poppy seeds at various volume fractions. We can compare their results with our data for cylindrical intruders into millet, the internal angle of which should be close to that of poppy seeds. From their data, we can estimate our $\tilde h$, $\tilde h_0$, and $\tilde p_u$. Translating our $\tilde h_0$ to their notations, using the different normalisation methods, we find that it corresponds to their *δ* = 3.8 mm, which is well within the range shown in the inset in their Fig. 3b. Specifically, using their fit in that inset, we get that this value corresponds to a packing fraction 0.600 ± 0.002. Comparing then the slope of their linear steady-state region in that packing fraction with our millet data, we find that it corresponds to our *K*_*ϕ*_ = 60 ± 2. Using now our relations (5) and (2), we obtain that internal friction angle of their medium should be 32.3° ± 0.3°, in excellent agreement both with the literature value and with the value we measured for our millet, 32° ± 2°. The wide range of values of *ϕ* in our tests covers most common granular materials, implying that our theoretical model has a broad application.

Equation (5) makes it also possible to derive the relation between *K*_*ϕ*_ and *μ*, which is shown in the inset of Fig. 5a. This relation is clearly superlinear, raising questions about the assumption of its linearity in the LFFM. The quality of the agreement between model and experiments also supports the prediction of Eq. (5) that *K*_*ϕ*_ is a constitutive property of the granular medium alone, an issue explored extensively in penetration experiments^5,23,34,37^.

### Modified Archimedes’ law in granular matter

Within the LFFM^23^, the resistance force arises from local forces acting normal to the intruder surface. Defining $- \widehat {\bf{z}}$ as the direction of gravity, with $\widehat {\bf{z}}$ a unit vector in the *z* direction, we determine the total resistance force on a convex intruder, advancing slowly in this direction, by using Eqs. (1) and (5), as well as Gauss law for inversion from a surface to a volume integral:

6 $$F_z = {\oint} {{\oint}_S {\widehat {\bf{z}} \cdot {\rm d}{\bf{F}}} } = K_\phi \rho _{\rm s}g{\int}_{V} {\rm div}\left( {h\widehat {\bf{z}}} \right){\rm d}V = K_\phi \rho _{\rm s}gV,$$

with *V* the volume of the displaced granular materials. This expression is equivalent to Archimedes’ law^44^ in fluid mechanics, in which *K*_*ϕ*_ = 1. The order-of-magnitude larger value of *K*_*ϕ*_ in granular media is due to an effective interaction of the intruder with the entire cyan region shown in Fig. 4b.

For flat-bottom intruders, Eq. (2) includes $\tilde p_0$, a constant term also observed by others^23,27^. As we show below, this is because the volume *V* in relation (6) should include the developing volume of the SZ, which gives rise to the initial nonlinear regime. We calculate this volume explicitly below.

**Fig. 6:**
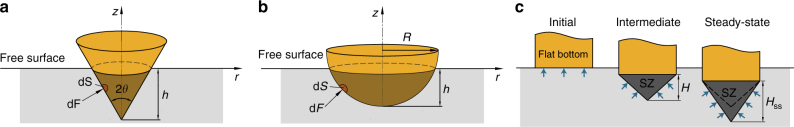
Illustration of the non-flat intruders and the growth of the SZ. **a** A conical intruder. **b** A semi-spherical intruder. **c** A sketch of the development of the SZ ahead of a flat-bottom intruder. Note the change in the directions of the normal forces as the SZ evolves

**Fig. 7:**
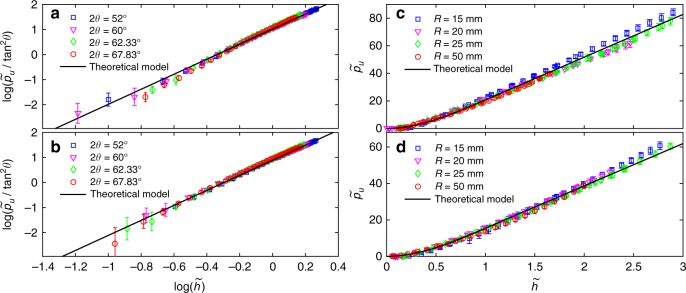
Intrusion of cones and spheres. **a**, **b** The rescaled force ${\mathrm{log}}\left( {\tilde p_u{\mathrm{/tan}}^{\mathrm{2}}\theta } \right)$ vs. ${\mathrm{log}}{\kern 1pt} \tilde h$, measured from the intruder’s bottom, for conical intruders of different head angles 2*θ* penetrating glass beads 2 and 3. We normalise depth by *R* = 20 mm to coincide with our cylindrical intruders experiments. The experimental data are in good agreement with the theoretical calculation (solid lines), $\tilde p_u = {\mathrm{tan}}^2{\kern 1pt} \theta K_\phi \tilde h^3{\mathrm{/}}3$. **c**, **d** The same as in **a**, **b** for spherical intruders of different radii, penetrating glass beads 2 and 3. Again, the experimental data agree well with the theoretical curves (solid lines): $\tilde p_u = K_\phi \tilde h^2(1 - \tilde h{\mathrm{/}}3)$ and $\tilde p_u = K_\phi (\tilde h - 1{\mathrm{/}}3)$ for $\tilde h \le 1$ and $\tilde h > 1$, respectively. All the measurements were taken on three samples for each of the four experiments, with the error bars representing the standard deviation over the samples. The fits qualities are: *R*^2^ >0.991, 0.993, 0.993 and 0.995, respectively, for **a**–**d**

For the non-flat intrusions (see Fig. 6), the quasistatic resistance force may be estimated directly from Eq. (6)^23^. We test four aluminium cones with head angles 2*θ* of 52°, 60°, 62.3°, and 67.8°, all to ±0.1°, and four aluminium spheres with radii of 15, 20, 25, and 50 mm, all to 0.01 mm, in two granular materials (glass beads 2–3). The experimental measurements are plotted in Fig. 7 and compared with the corresponding theoretical calculation of the resistance force from Eq. (6). The excellent agreement between all the theoretical and experimental results has two significant implications. One is that the quasistatic resistance force on a moving intruder satisfies Eq. (6). The other is that the agreement for all intruder shapes within a given medium with the same value of *K*_*ϕ*_ establishes that this parameter is a pure constitutive property of the granular medium. These conclusions can be used to derive theoretically the constant term in Eq. (2), which we proceed to do next.

### Prediction of $\tilde p_0$ and the SZ growth

Establishing the validity of the modified Archimedes’ law (6) for any intruder convex shape, allows us to derive the value of $\tilde p_0$, as well as the dynamic development of the SZ ahead of flat-bottom intruders. Consider an intruding prism of cross-section *S*, with a convex-shaped bottom of volume *V*_0_ and extension *h*_0_ in the penetration direction. For example, for a cylinder of radius $R{{\rm = }}\sqrt {S{{\rm /}}\pi }$ with either a half-spherical bottom or a cone of head angle 2*θ*, (*h*_0_, *V*_0_) = (*R*, 2π*R*^3^/3) or (*R*/tan*θ*, π*R*^3^/(3 tan*θ*)), respectively.

The intrusion volume is

7 $$V = V_0 + Sh,$$

where *h* is measured from the medium’s surface to the bottom of the linear part of the prism or cylinder. Substituting into the modified Archimedes’ law and rewriting it in terms of the normalised depth, $\tilde h = \left( {\sqrt {\pi {{\rm /}}S} } \right)h$, and pressure, $\tilde p_u = \sqrt \pi F_z{{\rm /}}\left( {\rho _{\rm s}gS^{3/2}} \right)$, we obtain

8 $$\tilde p_u = \tilde p_0 + K_\phi \tilde h = \frac{{\sqrt \pi K_\phi V_0}}{{S^{3/2}}} + K_\phi \tilde h.$$

Comparing to Eq. (2), the first term on the right-hand side is exactly $\tilde p_0$, which appears to depend on both the medium, through *K*_*ϕ*_, and the intruder shape parameters. Testing this result against measurements of conical and spherical intruders, we find an excellent agreement with the theory, as shown in Fig. 7.

Next, we apply this result to model the dynamic development of the SZ ahead of flat-bottom intruders. As discussed above, such intruders develop SZs that propagate ahead of them, parting the medium. The SZ buildup starts when the intruder enters the medium and ends once the SZ has reached an established steady-state shape. This dynamic process, illustrated in Fig. 6c, is the cause for the initial nonlinear dependence of $\tilde p_u$ on $\tilde h$.

To understand better this dynamic process, we model the developing SZ as an effective cone or polyhedron, whose apex is at $\tilde H = \left( {\sqrt {\pi {{\rm /}}S} } \right)H$ ahead of the intruder. Initially, *H* = 0 and it reaches a steady-state value, *H* = *H*_ss_ at the end of the nonlinear regime. The volume of this zone is *V*_0_ = *SH*/3 and, using Eq. (8), we have

9 $$\tilde H = \frac{{\left( {\tilde p_u{{\rm /}}K_\phi - \tilde h} \right)}}{3}.$$

Fitting now a functional form to *f*_1_(*h*) in Eq. (2), we can express $\tilde p_u$ in terms of $\tilde h$ in this regime, which provides the dependence of *H* on depth. Since the insertion of the intruder is at constant speed, this also provides the time evolution of the SZ.

In particular, we can find its steady-state size, $\tilde H_{{\rm ss}}$, for each granular material by substituting the crossover point values: $\tilde h = 0.15$ and $\tilde p_u\left( {\tilde h = 0.15} \right)$ (see Fig. 2). We find $\tilde H_{{\rm ss}}$ = 1.0 ± 0.1, 1.6 ± 0.3, 1.5 ± 0.2, 0.7 ± 0.3, and 3.1 ± 0.8 for glass beads types 1–3, sand, and millet, respectively. The error bars are the standard deviation of the measurements of $\tilde p_0$ in the different experiments.

## Discussion

In summary, we have proposed and tested a general model for the quasistatic penetration dynamics of convex solid objects into granular media. This is a key problem in granular science, relevant to diverse phenomena, such as animal and robotic locomotion^3–7^, crater formation^8–10^, granular flow dynamics^13,14^, and granular response to external loading^15–21^. A cone penetration test is also the standard for characterising soils.

First, by measuring experimentally the resistance forces on flat-bottom cylindrical and prismatic intruders, we find that all the force–depth curves can be generally collapsed onto a dimensionless pressure–depth master curve. This curve crosses over from a short initial nonlinear regime to a long steady-state linear one. Next, assuming a solid granular SZ, propagating ahead of the intruder, we construct a parameter-free model for the steady-state rate of the linear increase and show that this rate, *K*_*ϕ*_, depends only on the internal friction angle, *ϕ*, and is therefore a constitutive property of the granular medium. Our calculation fits all our observations, as well as all the reports we could find in the literature, which provided data we could compare with. Our results also call into question a conjecture in the literature that the gradient of the linear part is proportional to the internal friction coefficient^23^.

Then, using non-cylindrical intruders, cones and spheres, we show that the force–depth curves of all convex intruders satisfy a modified Archimedes’ law in that the resistance force is proportional to the hydrostatic-like pressure, the volume of the intruding object, and a proportionality coefficient *K*_*ϕ*_. The value of the latter is the only real difference between Archimedes’ law for ordinary fluids and dense granular matter. Significantly, the value of *K*_*ϕ*_ can be calculated theoretically, which renders our derived relation parameter-free! The validation of the model for the non-cylindrical intruders allowed us to derive the force–depth relation explicitly in terms of the medium’s constitutive property and the intruder’s geometry.

Specifically, in our model, the initial nonlinearity is a result of the growth process of the SZ, while the linear part corresponds to the SZ having reached a steady-state size, after which the effective volume of the intruder and the SZ does not change. This also explains the dependence of the gradient of the linear part only on the medium, since the resistance force is dominated by the normal force between the plastically flowing medium and the jammed SZ, both of which are made from the same material. This we have validated successfully by measurements on materials with internal frictional angles ranging from 22° to 36.5°.

Using this picture, we solve for the stress in the plastic zone around the SZ, using, for simplicity, the MC yield criterion model. This solution made it possible to calculate the total force on the intruder and the gradient *K*_*ϕ*_ explicitly as a function of *ϕ*. The solution agrees excellently with all the available experimental observations, ours and others’. Indeed, resistance force measurements on prisms of different cross-section shapes, confirm that *K*_*ϕ*_ is a constitutive property of the granular material alone.

The penetration may be affected strongly by the presence of container boundaries^30,31,33,34^, in particular by the side walls parallel to the intruder’s motion. Our theoretical model indicates that these effects arise from the interference between the stress field in the plastic zone and the walls. Theoretically, for avoiding such effects, the minimum container radius (MCR) is the horizontal extent of the longest characteristic, which is the green line in Fig. 4b, the length of which depends on *R* and *ϕ*. In Supplementary Table 1, we list the calculated MCR values for the five granular materials and four cylinder radii in our experiment. For some of the thick cylinders, it is larger than the container size we used. Nevertheless, the excellent collapse for all cases suggest no boundary effects. We conclude that the theoretical calculation, based on the MC criterion, provides an upper bound for the MCR. This is discussed in detail in Supplementary Note 3.

The modified Archimedes’ law also allows us to model quantitatively the SZ growth and predict its effective steady-state size. As a case study, we considered a flat-bottom cylinder, intruding at a constant speed below the critical value. On intruding, the volumes of both the immersed cylinder and a growing SZ, *V*_sz_, increase with depth. This gives rise to the initial nonlinear regime, in which the total volume increase is *V*(*h*) = *Sh* + *V*_sz_(*h*). *V*_sz_ increases slower than linearly until it reaches a steady-state value. Generally, assuming that the SZ is a cone throughout its growth (or pyramid-like for non-cylinders) of height *H*(*h*), we have *V*_sz_(*h*) = *SH*(*h*)/3, independent of the intruder’s cross-section shape. The inset in Fig. 3b displays a magnification of the nonlinear regimes for the different cross-sections. These all appear to collapse to a master curve, except when the cross-section aspect ratio is considerably different than one (see below).

Based on this insight, we used the nonlinear-to-linear crossover to estimate the steady-state height of the SZ. The errors of the estimates, for the different media, ranged from 10% to 40% and could stem from: (i) measurement errors of *ϕ*, which gives rise to errors in *K*_*ϕ*_ and $\tilde p_0$; (ii) error in estimating the nonlinear-to-linear transition point $\tilde h_0$; (iii) an inaccurate yield criterion in the initial nonlinear regime. The latter may also be the reason for an overestimate in the horizontal extent of the response region in the granular medium (cyan in Fig. 4). We also note that the SZ may be blunted at its edges and apex, making our estimates of its steady-state volume an upper bound.

To conclude, we have derived a parameter-free model for the penetration of solid objects into granular media, showing that the behaviour can be fully understood in terms of a modified Archimedes’ law. This understanding should shed more light on a wide range of practical issues, including the standard cone penetration test in soil mechanics in civil engineering, locomotion of animals or robots in sand, digging on Earth and extra-terrestrially, and crater formation, to name a few. It is also useful for furthering fundamental understanding of plastic flow of granular matter, e.g., in shear banding, the jamming transition, as well as bridging between the current different models for low- and high-velocity penetration regimes.

## Methods

### Experimental methods

Samples preparation: Before each penetration experiment, the granular medium was stirred by hand vigorously for 60 s. Then the free surface was flattened carefully by passing a trowel across it. Finally, two bubble levels were placed perpendicularly to one another to determine flatness of the surface. The same person prepared all samples to minimised personal variability. The penetration speed was controlled by a servo system to an accuracy of ±0.5% and displacement precision 0.001 mm. The resistance force was measured by a sensor to accuracy of ±0.5% and recorded by a software at sampling frequency of 20 Hz. The intruder was positioned about 3 mm above the flat surface and accelerated to reach a constant speed before it touched the granular surface. For flat-bottomed intruder, recording of the vertical resistance force and displacement started the instant the axial force exceeded 0.03 N, which was defined as the beginning of penetration. After checking that the data was independent of velocity (see Fig. 1), all the data was collected at an intrusion velocity of 30 mm min^−1^. Each experiment was carried out on a number of freshly prepared samples. The sample sizes for the experiments shown in Fig. 2, were: 13, 26, and 27, for the glass beads types 1–3, respectively, 26 for the sand, and 21 for the millet. For Fig. 3, we measured the intrusion force on three samples for each cross-section. The error bars represent the standard deviation over the samples and the fits qualities were: *R*^2^ >0.997, 0.992, 0.998, and 0.993, respectively, for the square, rectangle, equilateral triangle, and right-angle triangle. Similarly, the measurements on the non-cylindrical intruders, shown in Fig. 7, were taken on three samples for each of the four experiments, with the error bars in the figure also representing the standard deviation over the samples. The fits qualities in these cases were: *R*^2^ >0.991, 0.993, 0.993, and 0.995, respectively, for: (i) cones with four different head angles penetrating into glass beads type 2; (ii) same as (i) but into glass beads type 3; (iii) spheres of four different radii penetrating into glass beads type 2; (iv) same as (iii) but into glass beads type 3.

### Data processing

The experimental value of *K*_*ϕ*_ was determined by scaling the force–depth curves into a dimensionless form first and then using the first-order polyfit command in MATLAB to least-squares fit the linear slope. Figure 5 shows the results, with the points and error bars representing the mean and spread values of the slopes for the specific granular materials.

### Computation of *A*(*η*, *ϕ*) of Eq. (5)

The characteristic paths, shown in Fig. 4b, consist of a curved, *DE*, and linear, *CD*, parts, with the latter’s slope being tan*β*. Defining the distance from the cone apex along the path *OB* as *η* ∈ [0, 1], we have $l_0(\eta ) \equiv \overline {BE} = \frac{{(1 - \eta )R}}{{{{\rm sin}}{\kern 1pt} \beta }}$ and $l_\psi (\eta ) \equiv \overline {BJ} = l_0(\eta ){{\rm e}}^{\psi {\kern 1pt} {{\rm tan}}{\kern 1pt} \phi }$ (see Fig. 4). The positions of points *C*, *D*, *E* are:

10 $$\begin{array}{l}\left( {r_C,z_C} \right) = R\left( {1 + \frac{{2\left( {1 - \eta } \right)}}{{{{\rm tan}}{\kern 1pt} \beta }}{{\rm e}}^{\frac{\pi }{2}{{\rm tan}}{\kern 1pt} \phi },0} \right),\\ \left( {r_D,z_D} \right) = R\left( {1 + \frac{{R\left( {1 - \eta } \right)}}{{{{\rm tan}}{\kern 1pt} \beta }}{{\rm e}}^{\frac{\pi }{2}{{\rm tan}}{\kern 1pt} \phi },\left( {1 - \eta } \right){{\rm e}}^{\frac{\pi }{2}{{\rm tan}}{\kern 1pt} \phi }} \right),\\ \left( {r_E,z_E} \right) = R\left( {\eta ,\frac{-\eta }{{{{\rm tan}}{\kern 1pt} {\kern 1pt} \beta }}} \right).\end{array}$$

Focusing, for example, on the “+” family of Eq. (3), we have

11 $$\frac{{{\rm d}\sigma _0}}{{\sigma _0}} = - {{\rm tan}}{\kern 1pt} \phi \left( {2{\rm d}\alpha + \frac{{{{\rm cos}}{\kern 1pt} \phi }}{r}{\rm d}r + \frac{{1 - {{\rm sin}}\phi }}{r}{\rm d}z} \right)$$

and integrating Eq. (11) along the characteristic curve *CDE* from *C* to *E* gives

12 $${{\rm ln}}\left[ {\frac{{\sigma _{0,E}}}{{\sigma _{0,C}}}} \right] = {{\rm tan}}{\kern 1pt} \phi \left[ {\pi + {{\rm cos}}{\kern 1pt} \phi {\kern 1pt} {{\rm ln}}\frac{{r_C}}{{r_E}} + (1 - {{\rm sin}}\,\phi )\left( {{{\rm tan}}{\kern 1pt} \beta {\kern 1pt} {{\rm ln}}\frac{{r_C}}{{r_D}} + {\int}_{r_E}^{r_D} {\kern 1pt} \frac{{{\rm d}z}}{r}} \right)} \right].$$

The rightmost term in this relation can be calculated noting that: (i) the position of any point along the curve *DE* can be expressed in terms of variable *ψ*:

13 $$\left( {r(\psi ),z(\psi )} \right) = \left( {R + l_\psi (\eta ){{\rm sin}}(\psi - \beta ),-l_\psi (\eta ){{\rm cos}}(\psi - \beta )} \right)$$

and (ii) *ψ* ∈ [0, *π*/2] between points *D* and *E*:

14 $$Z \equiv {\int}_{r_E}^{r_D} \frac{{{\rm d}z}}{r} = {\int}_{0}^{\pi /2} \frac{{-(1 - \eta ){{\rm e}}^{\psi {\kern 1pt} {{\rm tan}}\phi }{{\rm cos}}(\psi + \beta )}}{{{{\rm cos}}\phi \left[ {{{\rm sin}}{\kern 1pt} \beta + (1 - \eta ){{\rm e}}^{\psi {\kern 1pt} {{\rm tan}}{\kern 1pt} \phi }{{\rm sin}}(\psi - \beta )} \right]}}{\rm d}\psi .$$

Substituting this into Eq. (12), we obtain finally for the mean stress at point *E*

15 $$\sigma _{0,E} = \sigma _{0,C}{\kern 1pt} {{\rm e}}^{\pi {\kern 1pt} {{\rm tan}}{\kern 1pt} \varphi }\left( {\frac{{r_C^{1 + {{\rm tan}}^{{\rm 2}}\beta }}}{{r_D^{{{\rm tan}}^{{\rm 2}}\beta }r_E}}} \right)^{{{\rm sin}}{\kern 1pt} \phi }{{\rm e}}^{{{\rm sin}}{\kern 1pt} \phi {\kern 1pt} {{\rm tan}}\beta Z} \equiv \sigma _{0,C}{\kern 1pt} {{\rm e}}^{\pi {\kern 1pt} {{\rm tan}}\,\phi }A(\eta ,\phi ),$$

where $A(\eta ,\phi ) \equiv \left( {\frac{{r_C^{1 + {{\rm tan}}^{{\rm 2}}\beta }}}{{r_D^{{{\rm tan}}^{{\rm 2}}\beta }r_E}}} \right)^{{{\rm sin}}{\kern 1pt} \phi }{{\rm e}}^{{{\rm sin}}{\kern 1pt} \phi {\kern 1pt} {{\rm tan}}{\kern 1pt} \beta Z}$ and $\sigma _{0,C} = \frac{{\rho _{\rm s}gh}}{{1 - {{\rm sin}}{\kern 1pt} \phi }}$, corresponding to the stress boundary condition at point *C*.

### Data availability

The data are available from the corresponding author on request.

## Electronic supplementary material

Supplementary Information(PDF 414 kb)

Peer Review File(TEX 28 kb)
