# Supplementary material for: Archimedes’ law explains penetration of solids into granular media
Source: Nat Commun. 2018 Mar 16;9:1101. doi: 10.1038/s41467-018-03344-3 (PMC5856792; doi:10.1038/s41467-018-03344-3)
Supplement: Supplementary file 1 — Supplementary Information(PDF 414 kb) [file 41467_2018_3344_MOESM1_ESM.pdf]

Supplementary Information

for

Archimedes' law explains penetration of solids into granular media

KANG ET AL.

Supplementary figures.

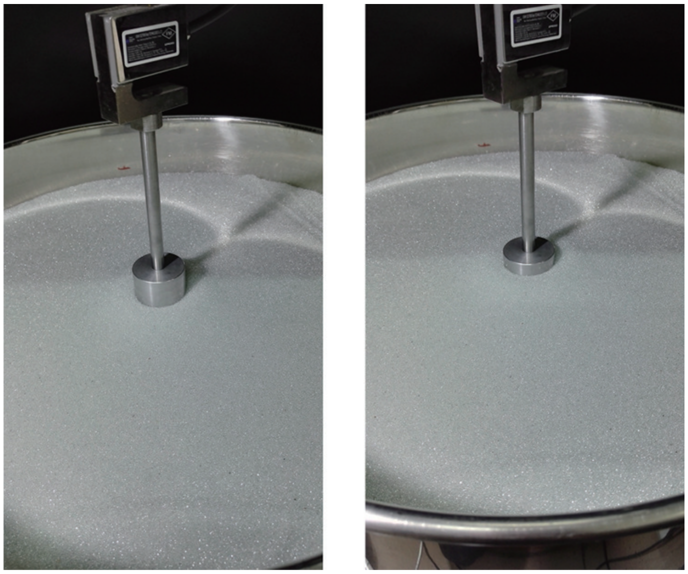

Supplementary Figure 1. The flatness of the granular surface during penetration. Experimental photos of the free surface at two stages during the penetration of a 20mm cylinder into type-1 glass beads. The photos substantiate that the deformations around the intruder, at two different depths, are indeed very small to the eye.

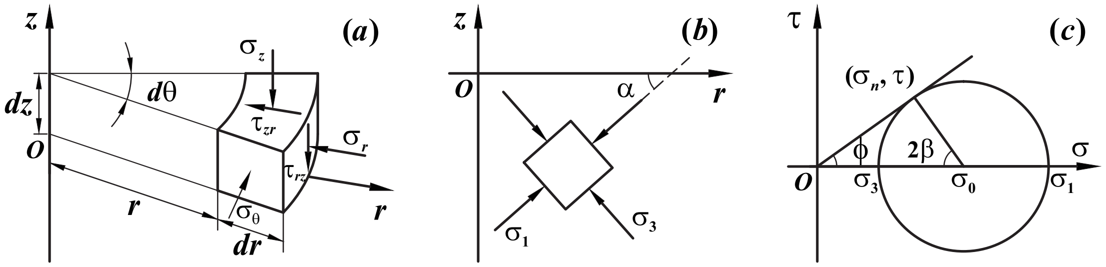

Supplementary Figure 2. The axi-symmetric stress components in cylindrical coordinates. We use the convention that all the vectors point in the positive direction. (a) The stress components on an infinitesimal material element. (b) The stress state in the $z-r$ plane; σ_1 and σ_3 are the major and minor principal stress, respectively, and α the angle between major principal stress and the radial direction. (c) Mohr's circle at incipient failure condition; σ_0 is the mean of the major and minor principal stresses.

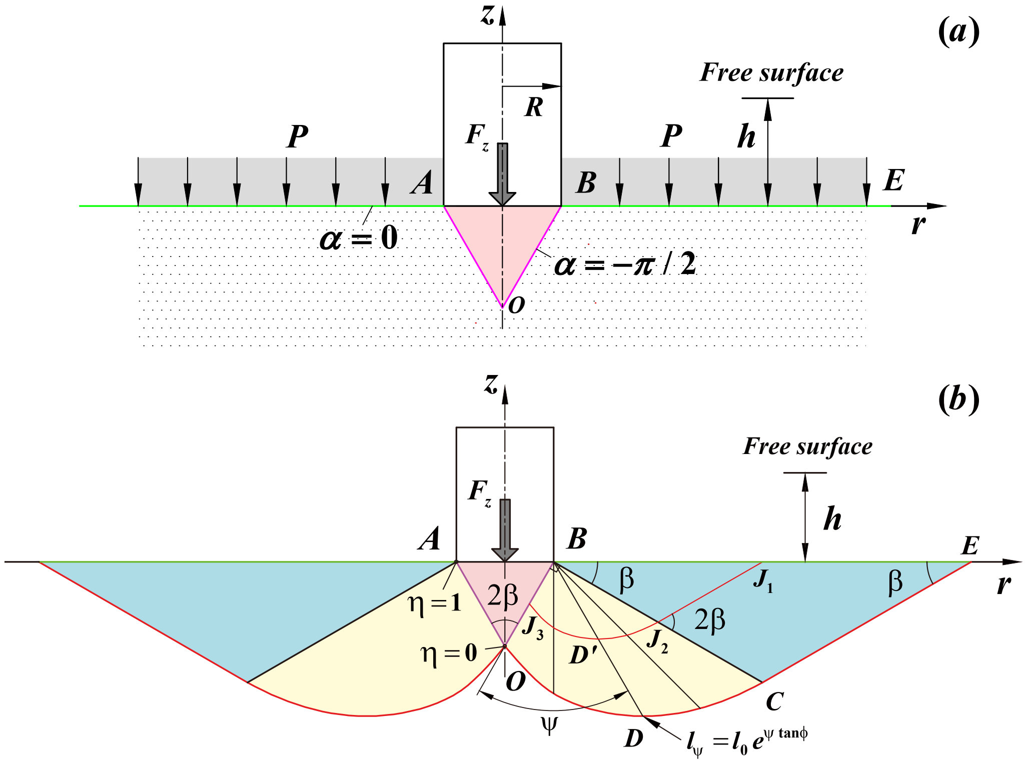

Supplementary Figure 3. The stress state around an intruding cylinder. (a) A sketch of the advancing cylinder and the stagnant cone ahead of it. A uniform hydrostatic-like pressure $P = \rho_s gh$ is presumed to act on the boundary BE. (b) The stress state in the plastic region: the angle $\beta = \pi/4 - \phi/2$ and the $+$ and $-$ characteristic curves in red and black, respectively. The plastic region consists of an active zone, AOB, a fan zone, OBC, and a passive zone, BCE. J_1, J_2, and J_3 are the intersection of an arbitrary $+$ curve with the lines BE, BC, BO, respectively.

Supplementary tables.

R (cm)	1.5	2	2.5	3.5
Glass beads 3 ($\phi = 26°$)	11.83	15.77	19.71	27.60
Glass beads 1 ($\phi = 27°$)	12.40	16.53	20.67	30.36
Glass beads 2 ($\phi = 28°$)	13.01	17.35	21.68	27.60
Millet ($\phi = 32°$)	15.94	21.26	26.57	37.20
Sand ($\phi = 36.5°$)	20.53	27.37	34.22	47.90

Supplementary Table 1. The horizontal extension of the stress response, $R_{\max}$. The value of $R_{\max}$, calculated from eq. (20) below, for the five granular materials and four intruding cylinders in our experiments. Shaded are the experiments, in which $R_{\max}$ is smaller than the system boundaries at $R_{sys} = 22.5$cm. Below, we discuss the significance of this comparison.

Supplementary notes.

Supplementary note 1. Method of characteristics.

Cohesionless dry granular media is usually modelled as a continuum of bulk density ρ_s and internal friction angle ϕ.[1] A fundamental concept in this description is a yield criterion[2] and a common such a criterion is the Mohr-Coulomb (MC):

$$|\tau/\sigma_n| = \mu \equiv \tan\phi \text{ or } \frac{\sigma_1 - \sigma_3}{2} = \frac{\sigma_1 + \sigma_3}{2} \sin\phi \ , \tag{1}$$

where τ is the local shear stress, σ_n is the corresponding normal stress and σ_1 and σ_3 are the major and minor principal stresses, respectively. Applying this criterion to the mechanical equilibrium conditions, yields a set of hyperbolic differential equations. These are solved by the method of characteristics.[3] The solution predicts the capacity of a shallow granular medium to support axisymmetric load.[4,5] In the following, we use this approach to calculate the quasistatic resistance force on an intruder, embedded at depth h. This allows us to derive K_ϕ in terms of ϕ.

The stress on a material element in cylindrical coordinates (r, θ, z) is shown in Supplementary Fig. 2a. The azimutal stress, σ_θ, is principal owing to the axial symmetry, i.e. $\tau_{\theta r} = \tau_{\theta z} = 0$. The major principal stress σ_1 and minor principal stress σ_3 are in the $z - r$ plane and, following Cox's assumption,[5] the intermediate stress is $\sigma_2 = \sigma_\theta = \sigma_3$. This enables us to analyse the plastic equilibrium by considering only the $z - r$ plane. Defining the mean principal stress in the $z - r$ plane, $\sigma_0 \equiv (\sigma_1 + \sigma_3)/2$, and the inclination angle between σ_1 and the radial direction, α (see Supplementary Fig. 2b), the stress components are:

$$\begin{aligned}
\sigma_r &= \sigma_0 + \frac{\sigma_1 - \sigma_3}{2} \cos 2\alpha \\
\sigma_z &= \sigma_0 - \frac{\sigma_1 - \sigma_3}{2} \cos 2\alpha \\
\tau_{rz} &= \frac{\sigma_1 - \sigma_3}{2} \sin 2\alpha \ .
\end{aligned} \tag{2}$$

The MC yield criterion is described by a straight envelope tangent to the Mohr circle in the principal stresses (Supplementary Fig. 2c). It follows that the stress components are functions of σ_0 and α:

$$\begin{aligned}
\sigma_r &= \sigma_0(1 + \sin\phi \cos 2\alpha) \\
\sigma_z &= \sigma_0(1 - \sin\phi \cos 2\alpha) \\
\sigma_\theta &= \sigma_0(1 - \sin\phi) \\
\tau_{rz} &= \sigma_0 \sin\phi \sin 2\alpha \ .
\end{aligned} \tag{3}$$

Neglecting gravity, the equations governing the equilibrium of the yield region are

$$\begin{aligned}
&\frac{\partial \sigma_r}{\partial r} + \frac{\partial \tau_{rz}}{\partial z} + \frac{\sigma_r - \sigma_\theta}{r} = 0 \\
&\frac{\partial \tau_{rz}}{\partial r} + \frac{\partial \sigma_z}{\partial z} + \frac{\tau_{rz}}{r} = 0 \ .
\end{aligned} \tag{4}$$

Defining

$$\mathbf{u} = \begin{pmatrix} \sigma_0 \\ \alpha \end{pmatrix} ,$$

and substituting from (3) into (4), the latter can be rewritten as

$$\mathbf{A}\cdot\partial_r\mathbf{u}+\mathbf{B}\cdot\partial_z\mathbf{u}=\mathbf{q}, \tag{5}$$

with

$$\mathbf{A}=\begin{pmatrix} 1+\sin\phi\cos 2\alpha & -2\sigma_0\sin\phi\sin 2\alpha \\ \sin\phi\sin 2\alpha & 2\sigma_0\sin\phi\cos 2\alpha \end{pmatrix},$$
$$\mathbf{B}=\begin{pmatrix} \sin\phi\sin 2\alpha & 2\sigma_0\sin\phi\cos 2\alpha \\ 1-\sin\phi\cos 2\alpha & 2\sigma_0\sin\phi\sin 2\alpha \end{pmatrix},$$
$$\mathbf{q}=-\frac{\sigma_0\sin\phi}{r}\begin{pmatrix} 1+\cos 2\alpha \\ \sin 2\alpha \end{pmatrix}.$$

Multiplying by $\mathbf{B}^{-1}$, we have

$$\left(\mathbf{B}^{-1}\mathbf{A}\right)\partial_r\mathbf{u}+\partial_z\mathbf{u}\equiv\mathbf{C}\partial_r\mathbf{u}+\partial_z\mathbf{u}=\mathbf{B}^{-1}\mathbf{q}\,. \tag{6}$$

Defining characteristic variables, $\omega_{1,2}$

$$\mathbf{u}=\mathbf{Y}\boldsymbol{\omega}=\begin{pmatrix} 1 & 1 \\ \lambda_1 & \lambda_2 \end{pmatrix}\begin{pmatrix} \omega_1 \\ \omega_2 \end{pmatrix}, \tag{7}$$

where $\lambda_{1,2}$ are the eigenvalues of $\mathbf{C}$ and $\mathbf{Y}^{-1}\mathbf{C}\mathbf{Y}=\boldsymbol{\Lambda}$ is diagonal, we can write (6) as

$$\partial_z\boldsymbol{\omega}+\boldsymbol{\Lambda}\partial_r\boldsymbol{\omega}=\mathbf{Y}^{-1}\mathbf{B}^{-1}\mathbf{q}-\mathbf{Y}^{-1}\left(\partial_z\mathbf{Y}+\mathbf{C}\partial_r\mathbf{Y}\right)\boldsymbol{\omega}\,. \tag{8}$$

It is now convenient to parameterise the lengths of the characteristic paths by s_i: $\partial_z s_i=1$ and $\partial_r s_i=1/\lambda_i$ $(i=1,2)$, in terms of which eqs. (8) decouple:

$$\frac{d\boldsymbol{\omega}}{ds}=\mathbf{Y}^{-1}\mathbf{B}^{-1}\mathbf{q}+\mathbf{E}, \tag{9}$$

with $\mathbf{E}=-\mathbf{Y}^{-1}\left(\partial_z\mathbf{Y}+\mathbf{C}\partial_r\mathbf{Y}\right)\boldsymbol{\omega}$.
The solution to (9) yields the slopes of two families of characteristic curves,

$$\kappa_\pm\equiv dz/dr=1/\lambda_i=\tan\left(\alpha\pm\beta\right)\quad;\quad\beta=\pi/4-\phi/2. \tag{10}$$

We name the two families of characteristic curves $+$ and $-$ and note that the angle between them, at any one point in the $r-z$ plane, is always 2β. Along the characteristics, the decoupled equations are:

$$d\sigma_0=-\sigma_0\tan\phi\left(\pm 2d\alpha+\frac{\cos\phi}{r}dr\pm\frac{1-\sin\phi}{r}dz\right)\,. \tag{11}$$

Together with stress boundary conditions, these determine the stress state in the plastic region.
We assume that the top free surface, indicated in Supplementary Fig. 3a, remains flat as the intruder advances quasistatically into the medium. This was supported by observations that the deformations to that surface around the intruder, at two different depths,

were much smaller than the estimated stagnant zone size (see below). The boundary conditions are: a) a resistance force on the cylinder bottom AB, leading to a resultant axial force $F_z = K_\phi S \rho_s g h$; b) a uniform hydrostatic-like pressure, $P = \rho_s g h$, on the horizontal plane BE, level with the bottom of the cylinder AB. Any shear stress on boundaries AB and BE is neglected relative to the normal forces on the advancing intruder. From eq. (3), we obtain that, at the two boundaries, $\alpha = 0$ or $\pm\pi/2$, while at AB and BE we have $\alpha_{AB} = -\pi/2$ and $\alpha_{BE} = 0$. The geometry of the plastic zone is cylindrically symmetric and, as shown in Supplementary Fig. 3b, it consists of three regions: a triangular passive zone, BCE; a triangular active zone, AOB, with an apex angle 2β; a fan-shaped transition zone, OBC, of sector angle $\pi/2$ and a contour line satisfying $l_{\psi B} = \overline{BD} = l_0 \mathrm{e}^{\psi \tan\phi}$ and $l_0 = \overline{BO} = R/\sin\beta$.

Supplementary note 2. Calculation of K_ϕ.

Experimental measurements on the planar granular flow field around a vertically slowly moving finger[6,7] and a flat-ended punch[8,9] support the shape of yield region shown in Supplementary Fig. 3b. Those experiments also confirmed the existence of a stagnant zone (SZ) ahead of the advancing object, in which the granular medium moves as a rigid body with the intruder. Based on previous studies,[8,10] we assume that the SZ has a conical shape, with an apex angle, AOB, that is a function of the internal friction angle ϕ. The formation of the SZ is a result of local shear jamming.[11]

Thus, the conical SZ acts as a boundary, at which the characteristic curves initiate. We then integrate (11) along an arbitrary + characteristic curve, e.g. $J_1J_2J_3$ in Supplementary Fig. 3b, each of which consists of a straight part, e.g. $J_1 - J_2$, of slope of $\tan\beta$, and a curved part, e.g. $J_2 - J_3$. We then have

$$\begin{aligned} l_0(\eta) &= \overline{BJ_3} = \frac{(1-\eta)R}{\sin\beta} \\ l_\psi(\eta) &= \overline{BD'} = l_0(\eta)\mathrm{e}^{\psi\tan\phi} , \end{aligned}$$

where $\eta = \overline{OJ_3}/\overline{OB} \in [0,1]$, and the variables l_0, ψ and l_ψ are defined in Supplementary Fig. 3b. The (r,z) coordinates of point J_1, J_2 and J_3 are then: $R\left(1+\frac{2(1-\eta)}{\tan\beta}\mathrm{e}^{\frac{\pi}{2}\tan\phi},0\right)$, $R\left(1+\frac{(1-\eta)}{\tan\beta}\mathrm{e}^{\frac{\pi}{2}\tan\phi},-(1-\eta)\mathrm{e}^{\frac{\pi}{2}\tan\phi}\right)$, and $R\left(\eta,-\frac{\eta}{\tan\beta}\right)$, respectively.

For the + family of (11), we get

$$\frac{d\sigma_0}{\sigma_0} = -\tan\phi(2d\alpha + \frac{\cos\phi}{r}dr + \frac{1-\sin\phi}{r}dz) \tag{12}$$

and integrating this expression along the characteristic curve $J_1J_2J_3$ from J_1 to J_3, gives

$$\ln\left[\frac{\sigma_0(\eta)_{J_3}}{\sigma_0(\eta)_{J_1}}\right] = \tan\phi\left[\pi + \cos\phi\ln\frac{r_1}{r_3} + (1-\sin\phi)\left(\tan\beta\ln\frac{r_1}{r_2} + \int_{r_3}^{r_2}\frac{dz}{r}\right)\right] . \tag{13}$$

Note that the points along the curve $J_2 - J_3$ can be parametrised by ψ: $(r(\psi),z(\psi)) = \left(R + l_\psi(\eta)\sin(\psi-\beta), -l_\psi(\eta)\cos(\psi-\beta)\right)$. Since $\psi \in [0,\pi/2]$ between points J_2 and

J_3, we have

$$\begin{aligned} Z(\eta,\phi) &\equiv \int_{r_3}^{r_2} \frac{dz}{r} = \int_{r_3}^{r_2} \frac{d\left(-l_\psi(\eta)\cos(\psi-\beta)\right)}{R+l_\psi(\eta)\sin(\psi-\beta)} \\ &= \int_0^{\pi/2} \frac{-(1-\eta)\mathrm{e}^{\psi\tan\phi}\cos(\psi+\beta)}{\cos\phi\left[\sin\beta+(1-\eta)\mathrm{e}^{\psi\tan\phi}\sin(\psi-\beta)\right]} d\psi \, . \end{aligned} \tag{14}$$

Substituting this expression into (13), we obtain the mean stress at point J_3:

$$\begin{aligned} \sigma_0(\eta)_{J_3} &= \sigma_0(\eta)_{J_1}\mathrm{e}^{\pi\tan\phi}\left(\frac{r_1^{1+\tan^2\beta}}{r_2^{\tan^2\beta}r_3}\right)^{\sin\phi} \mathrm{e}^{\sin\phi\tan\beta Z(\eta,\phi)} \\ &\equiv \frac{P}{1-\sin\phi}A(\eta,\phi)\mathrm{e}^{\pi\tan\phi} \, , \end{aligned} \tag{15}$$

where $A(\eta,\phi) \equiv \left(\frac{r_1^{1+\tan^2\beta}}{r_2^{\tan^2\beta}r_3}\right)^{\sin\phi} \mathrm{e}^{\sin\phi\tan\beta Z(\eta,\phi)}$. Similarly, the distribution of σ_z along the surface OB is obtained from the relationship between σ_z and σ_0,

$$\sigma_z(\eta) = (1+\sin\phi)\sigma_0(\eta) \, . \tag{16}$$

The resistance force on the SZ AOB is

$$F_z = \oiint_{cone} \sigma_{\mathbf{z}}(\eta)\cdot d\mathbf{S} \tag{17}$$

and, combining this with (16) gives,

$$F_z = \rho_s gh\left(2\pi R^2\frac{1+\sin\phi}{1-\sin\phi}\mathrm{e}^{\pi\tan\phi}\int_0^1 \eta A(\eta,\phi)d\eta\right) . \tag{18}$$

It follows that the coefficient of $\rho_s gSh$, defined in the text as K_ϕ, is

$$K_\phi = \frac{2(1+\sin\phi)}{1-\sin\phi}\mathrm{e}^{\pi\tan\phi}\int_0^1 \eta A(\eta,\phi)d\eta \, . \tag{19}$$

Thus, the material ‘constitutive’ parameter K_ϕ depends only on the internal friction angle ϕ.

Supplementary note 3. The horizontal extension of the stress response.

Assuming that the SZ forms a perfect cone ahead of a cylindrical intruder of radius R, the horizontal extension of the stress response corresponds to the longest characteristic, namely, the one forming the boundary of the cyan region in Supplementary Fig. 3b. Its horizontal extent, $R_{\max}$, can be calculated from the above stress equations, yielding

$$R_{\max} = \left(1+\frac{2}{\tan(\pi/4-\phi/2)}\mathrm{e}^{\frac{\pi}{2}\tan\phi}\right)R. \tag{20}$$

Thus, under the Mohr-Coulomb criterion, $R_{\max}/R$ depends only on the internal friction angle ϕ, increasing with it. For the five granular materials we tested, $26° \leq \phi \leq 36.5°$, for which eq. (20) gives $7.9 \leq R_{\max}/R \leq 13.7$. The specific values of $R_{\max}$ for each experiment are given in the supplementary table 1. These values require some discussion.

In principle, one expects boundary effects to be negligible when $R_{\max}$ is smaller than the container size, $R_{\text{sys}} = 22.5$cm. Therefore, one could argue that this is not the case with increasing ϕ for some of the thick cylinders, as shown in the supplementary table 1. Yet, our results seem to be unaffected by boundary effects in all our experiments. We argue that eq. (20) gives an upper bound for $R_{\max}$, and apparently not a tight one. This argument rests on three reasons. Firstly, and most compellingly, given the granular medium, the steady-state force-depth curves collapse nicely to a single master curve for all intruder sizes. Thus, all the intruders, whether thick or thin, experience the same boundary effects. It follows that, if the thinest intruder is unaffected by the boundary, then neither are the thicker ones. Secondly, the longest characteristic, the cyan line in supplementary Fig. 3b, emanates from the apex of the ideal conical SZ. However, in reality, we expect the apex to blunt into a small curved surface, thus reducing the extent of $R_{\max}$. Thirdly, other results in the literature established that side wall effects can be neglected when $R_{\text{sys}} \geq 5R$,[12] which is always the case in our experiments.

Supplementary References

1. Askari, H. & Kamrin, K. Intrusion rheology in grains and other flowable materials. Nat. Mater. (2016).
2. Michalowski, R. An estimate of the influence of soil weight on bearing capacity using limit analysis. Soils and Found. **37**, 57–64 (1997).
3. Sokolovski, V. Statics of soil media (1960).
4. Ibsen, L. B., Barari, A. & Larsen, K. A. Modified vertical bearing capacity for circular foundations in sand using reduced friction angle. Ocean Eng. **47**, 1–6 (2012).
5. Cox, A. D., Eason, G. & Hopkins, H. G. Axially symmetric plastic deformations in soils. Philos. Trans. R. Soc. London A: Math., Phys. and Eng. Sci. **254**, 1–45 (1961).
6. Hamm, E., Tapia, F. & Melo, F. Dynamics of shear bands in a dense granular material forced by a slowly moving rigid body. Phys. Rev. E **84**, 041304 (2011).
7. Tapia, F., Espíndola, D., Hamm, E. & Melo, F. Effect of packing fraction on shear band formation in a granular material forced by a penetrometer. Phys. Rev. E **87**, 014201 (2013).
8. Murthy, T. G., Gnanamanickam, E. & Chandrasekar, S. Deformation field in indentation of a granular ensemble. Phys. Rev. E **85**, 061306 (2012).
9. Viswanathan, K., Mahato, A., Murthy, T. G., Koziara, T. & Chandrasekar, S. Kinematic flow patterns in slow deformation of a dense granular material. Granul. Matter **17**, 553–565 (2015).
10. Aguilar, J. & Goldman, D. I. Robophysical study of jumping dynamics on granular media. Nat. Phys. (2015).
11. Bi, D., Zhang, J., Chakraborty, B. & Behringer, R. P. Jamming by shear. Nature **480**, 355–358 (2011).

12. Seguin, A., Bertho, Y. & Gondret, P. Influence of confinement on granular penetration by impact. Phys. Rev. E **78**, 010301 (2008).
